# **Synergistic antifungal and anti-aflatoxigenic effects of lactic acid bacteria and golden berry in a functional high-protein beverage**

**DOI:** 10.1038/s41598-025-24160-y

**Published:** 2025-12-08

**Authors:** Tarek Nour Soliman, Ahmed Noah Badr, Feriala A. A. Abu Safe, Karolina Hoppe, Wayne T. Shier, Yehia Hassan Abu Sree

**Affiliations:** 1 Dairy Department, Food Industries and Nutrition Research Institute, National Research Centre, Dokki, Cairo, 12622 Egypt; 2 Food Toxicology and Contaminants Department, National Research Centre, Dokki, Cairo, 12622 Egypt; 3 Academy of Scientific Research & Technology (ASRT), 101 Kasr Al-Ainy St, Cairo, Egypt; 4 Botany Department, Faculty of Women for Arts, Science, and Education, Ain Shams University, Cairo, Egypt; 5 Department of Chemistry, Poznań University of Life Science, ul. Wojska Polskiego 75, 60- 625 Poznań, Poland; 6 Medicinal Chemistry Department, Minnesota University, Minneapolis, MN 55455 USA

**Keywords:** Fermented high-protein beverage, Lactic acid bacteria, Anti-aflatoxigenic, Antifungal activity, High-protein beverage characteristics, Biotechnology, Microbiology

## Abstract

High-protein beverages are valued for their health benefits, but they face threats from toxigenic fungi and mycotoxins This study developed a novel, functional high-protein beverage (HPB) by synergistically combining whey protein concentrate (WPC), fermented by specific bacteria, with *Physalis peruviana* (golden berry) powder (at a 1:5; w/w). Three bacterial strains—*Lactobacillus plantarum* (BS-2), *L. pentosus* (BS-1), and *L. paracasei* (BS-3)—were involved for the fermentation, individually and in combinations. Fortification with golden berry significantly enhanced the phytochemical profile, increasing phenolic content (22.97 ± 0.56 mg GAE/g), flavonoids (97.15 ± 2.58 mg QE/g), and antioxidant capacity compared to the control. The BS-2 strain exhibited the strongest antifungal activity, inhibiting fungal growth by 53.8 ± 0.41% and completely suppressing the aflatoxin B_2_ production by *Aspergillus parasiticus*. Rheological analysis showed that BS-2 and the three-strain mixture significantly increased viscosity (*p* < 0.05), enhancing beverage stability. Sensory evaluation (*n* = 40) revealed that the BS-2 fermented *Physalis*-HPB was the most preferred, earning the highest overall score. During 21-day shelf-life tests under fungal challenge, beverages fermented with BS-2 and mixed strains maintained the lowest fungal CFU counts at 4 °C, demonstrating superior microbial stability. These findings show that combining *P. peruviana* with probiotic fermentation enhances the functional, sensory, and safety qualities of HPBs, suggesting a promising bio-based approach to reduce fungal spoilage and mycotoxin hazards in dairy beverages.

**Supplementary Information:**

The online version contains supplementary material available at 10.1038/s41598-025-24160-y.

## Introduction

Consumer demand for natural, health-promoting products drives the development of functional foods with enhanced nutritional and protective properties^1,2^. High-protein beverages (HPBs), especially those based on whey protein concentrate (WPC), are effective vehicles for delivering bioactive peptides, essential amino acids, and immunomodulatory compounds that support muscle synthesis, metabolic regulation, and gut health^3,4^. Despite their nutritional value, HPBs are susceptible to microbial spoilage. Contamination by toxigenic fungi, which produce harmful secondary metabolites like aflatoxins, is a significant concern^5,6^. Aflatoxins, classified as Group 1 carcinogens by the International Agency for Research on Cancer (IARC), pose a major global food safety risk^7^. Their presence in high-moisture, protein-rich matrices like dairy beverages raises serious public health and regulatory concerns.

Fermentation with lactic acid bacteria (LAB) offers a viable bio-preservation strategy to mitigate fungal proliferation and aflatoxin production. LAB are well-documented for their antifungal properties, mediated through the secretion of organic acids, bacteriocins, hydrogen peroxide, and low-molecular-weight antifungal peptides^8,9^. Specific Lactobacillus strains can inhibit mycotoxigenic fungi through direct antagonism and mycotoxin-binding mechanisms^10,11^. Using bacterial cell-free supernatants provides a potentially safer antifungal approach, as it avoids introducing viable cells. This is particularly advantageous for non-cultured or shelf-stable beverages.

The integration of plant-based bioactives has emerged as a complementary or synergistic approach to enhance the functional and preservative qualities of dairy beverages. *Physalis peruviana* L., or golden berry, is a fruit abundant in polyphenols, flavonoids, carotenoids, and antioxidant compounds, which are associated with anti-inflammatory, antimicrobial, and anticarcinogenic properties^12,13^. Recent studies demonstrate that golden berry extracts possess significant antifungal and free-radical scavenging .properties, indicating potential for food stabilization and improved nutraceutical quality^14,15^. However, its application in protein-rich fermented systems, particularly its effects on fungal inhibition, aflatoxin suppression, and sensory properties, remains underexplored.

This study aimed to develop a fermented high-protein beverage from WPC using specific probiotic LAB strains (*Lactobacillus plantarum*, *L. pentosus*, and *L. paracasei*) fortified with golden berry powder. It was evaluated the beverage’s phytochemical content (phenolics, flavonoids), antifungal efficacy, and ability to inhibit aflatoxin B_1_ (AFB_1_) production. It was also conducted sensory analysis and viscosity measurements to assess product acceptability, and performed fungal inoculation assays to determine the impact of fermentation and fortification on shelf-life extension. The study presents a new method that combines microbial fermentation with natural phytochemical enrichment to produce a safe, long-lasting, and nutritious high-protein beverage.

## Materials and methods

### Materials

#### Applied materials

The WPC and glucose syrup (GS) were purchased from Alfasol Co., Turkey. Fresh *Physalis peruviana* fruits were blended (Munro LB20ES, UK) and lyophilized (Esquire Biotech FD-10-MR, India) to produce the powder used in this study.

#### Chemicals

All Chemicals and media were purchased from Sigma-Aldrich, St. Louis, MO 68178, USA. These chemicals are Folin-Ciocalteu reagent; 2,2′-Azino-bis(3-ethyl benzothiazole-6-sulfonic acid (ABTS^+^); 6-hydroxy-2,5,7,8-tetramethyl chroman-2-carboxylic acid (Trolox^®^); aflatoxins standards; aluminum chloride; sodium nitrate; analytical grade solvents. The microbial growth media were De-Man Rogosa and Sharpe (MRS), Yeast extract sucrose (YES), malt extract agar (MEA), and potato dextrose agar (PDA).

#### Microorganisms

The starter bacterial strains, previously isolated from local products and identified by Sakr et al., were selected for their capacity to produce oligo- and polysaccharides, which can enhance the fermentation process^16^. It was reactivated twice on De-Man Rogosa and Sharpe (MRS) broth before being utilized for fermentation. These strains were *Lactobacillus pentosus* MK 616,468, *Lactobacillus plantarum* MK 616,469, and *Lactobacillus paracasei* MT 383,743. A bacterial starter applied for the positive control fermentation were *Streptococcus thermophiles* BAA-250 and *Lactobacillus delbrueckii subsp. bulgaricus* ATCC 11,842. These strains were taken as gifts from the dairy Department, the NRC. Lyophilized starters were reactivated separately (0.02% w/v inoculum) in autoclaved skim milk (0.09–0.02% fat; 10% SNF; 110 °C/15 min). The activated cultures were then used to ferment the high-protein milk beverage. Fungi strains used for antifungal evaluations were *Aspergillus flavus* ITEM 698, *A. niger* ITEM 7097, *A. carbonarius* ITEM 5010, *and A. parasiticus* ITEM 11, purchased from the agro-food microbial culture collection, ISPA, CNR, Italy. Lyophilized Fungal strains were reactivated twice on the PDA media using prepared spore suspension of individual fungal spores.

### Preparation of the fermented high-protein beverage

The WPC was utilized to prepare the HPD (with/without *Physalis peruviana*). The fermented high-protein beverage was made from the WPC of 80% (w/v) with a glucose syrup of 5% (w/v). Then, dried *Physalis peruviana* powder was added in a ratio (1:5) of the dry weight of the WPC using the same processing methods as shown in Table 1. One or more of the three starters (1, 2, and 3) were used for inoculation for fermentation (Table 1).

**Table 1:** Rrecipe of the ingredients content used for beverage preparations mixes.

No.	WPC (%)	GB (%)	GS (%)	Starter strain	No.	WPC (%)	GB (%)	GS (%)	Starter strain
T1	0	0	0	Control	T5	20	5	5	S1 + S2
T2	20	5	5	S1	T6	20	5	5	S1 + S3
T3	20	5	5	S2	T7	20	5	5	S2 + S3
T4	20	5	5	S3	T8	20	5	5	S1 + S2 + S3

S1:bacterial strain *Lactobacillus pentosus* strain S2: *Lactobacillus plantarum* strain; S3: *Lactobacillus paracasei;* Control: commercial fermented beverage fermented using *Streptococcus thermophiles* BAA-250 and *Lactobacillus delbrueckii* subsp. *bulgaricus* ATCC 11,842; WPC: whey protein concentrate; GB: goldern berry (dried powder); GS: glucose syrup.

### Determination of chemical composition

The Chemical compositions of WPC, *Physalis peruviana* powder, the high-protein beverage, and the high-protein beverage with *Physalis* were determined according to the AOAC guidelines described before^17^.

### Determination of total phenolic and total flavonoid contents

Phenolic compounds were extracted and total phenolic content (TPC) was determined as described previously^18^. Briefly, 0.5 mL of sample was mixed with 0.5 mL of 10-fold diluted Folin-Ciocalteu reagent. After 5 min, 4 mL of sodium carbonate solution (7.5%) was added. The mixture was incubated in the dark for 30 min at 25 °C, and absorbance was measured at 765 nm using a spectrophotometer (Model 1700, UVISON Technologies, Wrotham Sevenoaks, UK). The results were expressed in milligrams of Gallic acid equivalent (GAE) per gram of dry weight (DW).

Total flavonoid content (TFC) was determined using a colorimetric assay^13^. Briefly, 1.5 mL of extract was mixed with 75 µL of NaNO₂ (5%), incubated for 6 min, followed by addition of 150 µL of AlCl₃ (10%) and another 6 min incubation. Then, 0.5 mL of 1 M NaOH was added, and the volume was adjusted to 2.5 mL with water. Absorbance was measured at 510 nm. TFC was calculated from a quercetin standard curve and expressed as mg QE/g DW. For the blank, the extract was replaced with deionized water. The standard calibration curve was generated with concentrations of 0.01, 0.05, 0.1, 0.2, and 0.4 mg quercetin/mL. The total flavonoid concentration was calculated as mg equivalents of quercetin (QE) per gram of DW.

### Determination of antioxidant activity using ABTS^+^ assay

The total antioxidant capacity of samples was evaluated using the ABTS^**+**^ radical scavenging assay according to the technique described before^19^. Briefly, Potassium persulfate (2.6 mM) was added to an aqueous solution of ABTS^+^ (7 mM) to generate a stock solution of ABTS^+^ radicals, which was stored in the dark (25℃ /16 h). The stock solution was diluted with methanol, and samples (0.3 mL) were added to the working solution (2.7 mL). The mixtures were incubated (30 min/ 25 ℃) and then centrifuged (12,000 g /2 min /25 ℃). Against the blank, the absorbance values were determined using a spectrophotometer (734 nm). The results were expressed as milligram Trolox equivalents (TE) per gram of the beverage.

### Determination of probiotic strain antifungal activity

The antifungal activity of the probiotic bacterial starters against mycotoxigenic fungi (*A. flavus*,* A. niger*,* A. carbonarius*,* A. parasiticus*) was assessed^20^. Fungal strains were cultured on MEA at 25 °C. Conidia were harvested in a Tween-saline solution (0.9% NaCl, 0.2% Tween 80), and spore concentration was adjusted to 10^3^/mL. Bacterial strains were grown in MRS broth (37 °C, 24 h) to late log phase, spot-inoculated onto MRS agar plates, and incubated (30 °C, 5 days). The plates were then overlaid with soft MEA (0.7%, 9 mL) containing 10³ spores/mL of the target fungus. After incubation (25 °C, 5 days), inhibitory zones around bacterial colonies were measured (mm).

### Estimation of the antifungal activity for bacterial supernatants

The antifungal impact of the probiotic strains^11^, their extracts^21^, and bacterial supernatant^22^ have been mentioned earlier. The probiotic bacteria strains were grown in MRS broth (16 h/ 37 °C). Fermented supernatants were centrifuged (4000 rpm, 15 min) and filter-sterilized (0.22 μm) to ensure only metabolites, not live bacteria, were tested in antifungal and aflatoxin assays. The spores of each investigated fungus were plated (10^3^ spores /mL) on the PDA agar. The wells (5 mm diameter) were formed in the media agar plates utilizing a sterile cork-borer tool, which were filled with 100 µL of the supernatants and then incubated (25 °C/72 h). Inhibition zones surrounding the wells on the plates were analyzed and expressed in millimeters in diameter.

### Anti-aflatoxigenic properties of bacterial supernatants

The effect of the extracted supernatant for the three applied strains of bacteria: *Lactobacillus pentosus*, *Lactobacillus plantarum*, and *Lactobacillus paracasei*, was determined as described before. The anti-aflatoxigenic activity of cell-free supernatants from the three LAB strains was evaluated against *A. parasiticus* ITEM 11. A spore suspension (1.2–1.4 × 10³ CFU/mL) in Tween-saline was prepared. For fungal inhibition assessment, 100µL of each supernatant was added to YES broth inoculated with the spore suspension; a control flask received 100 µL of sterile water. Flasks were incubated (at 25 °C / 5 days). The mycelia were then filtered, dried to constant weight (45 °C, 24 h), and the inhibition ratio was calculated relative to the control. All procedures were performed under a laminar flow hood to maintain sterility. Control flasks containing YES media with 100µL of autoclaved, sterile water instead of bacterial supernatant were prepared and handled identically to the treatment flasks to account for any potential contamination.

For aflatoxin reduction assessment, a separate set of flasks was incubated at 28 °C for 12 days. The culture filtrate was passed through a 0.45 μm syringe filter and purified using an Afla-test^®^ column (Vicam). Aflatoxin content was quantified using a fluorimeter calibrated to 1.0 ng/L^23^.

### Determination of high-protein beverage viscosity

The apparent viscosity of the fermented WPC beverage was measured with a Bohlin coaxial cylinder viscometer (Bohlin Instrument Inc., Sweden) that was joined to a workstation running software (Viscometer program V88)^24^. The viscometer probe (system C30) was placed in the beverage sample cup, and viscosity measurements were carried out at 20 °C ± 2 °C in the up mode at shear rates ranging from 37 to 1238 1/s.

### Determination of physical, chemical, and sensory attributes

Standard AOAC methods that described by Kiros et al.^25^ were used to assess *physical* (pH, acidity) and chemical parameters (total solids, protein, fat, ash). Total carbohydrates were calculated by difference. A sensory evaluation was conducted to determine if the enhanced functional properties translated to consumer acceptability. The sensory evaluation was performed by 40 panelists (25 female, 15 male), aged between 25 and 55 years, all of whom were researcher staff from the Dairy and Food Science Departments, National Research Centre (NRC), with experience in dairy product evaluation. Panelists were selected based on availability and familiarity with fermented dairy products. Panelists were selected based on their availability, familiarity with fermented dairy products, and lack of known taste or smell disorders. Prior to evaluation, a training session was conducted to familiarize the panelists with the scorecard and the attributes to be assessed (color, flavor, taste, texture, consistency, and overall acceptability), as described before^26^.

### Determination of the shelf-life extension

The amelioration impact of the bacterial starters was measured according to the previous studies of Kumar et al.^27^, with slight modification. The high-protein beverage samples were saved in a sealed sterile container; each beverage type was kept individually for the three starters. Each container was swab-inserted using a cotton swab inoculated with a spore suspension (1 mL) of *A. parasiticus* (10^3^ CFU/mL). The negative control sample was stored under the same conditions as the sample free from fungal inoculation. The positive control was prepared without bacterial strains, and the spore suspension was inoculated into the container. The containers were stored in a refrigerator (4 ℃/21 days), and the number of fungi in each sample was determined on every odd day of the experiment.

### Statistical analysis

Statistical analysis was evaluated using the Statistical Analysis Software System for Windows (SPSS; Ver 16). Analysis of variance (ANOVA) was utilized to assess the significant difference between the mean values, and Duncan’s multiple range test was done at a significance level of *p* < 0.05. All samples were examined in triplicate, and results were expressed as means ± standard error mean (SEM).

## Results and discussion

### Chemical composition of raw and processed materials

The chemical composition of the raw materials and the final fortified beverage is shown in Table 2. WPC had a high protein content (79.86 ± 0.14%), while *Physalis* powder was rich in carbohydrates (65.21 ± 1.27%). The fortified HPB showed a superior nutritional profile compared to the plain beverage, with higher protein (16.17 ± 0.23%), carbohydrate, and ash content. These differences highlight the synergistic nutritional enhancement achieved by *Physalis* fortification, consistent with previous studies on incorporating plant bioactives into dairy beverages^1,24,28,29^.

**Table 2:** Chemical composition of Whey protein concentrate, lyophilized *Physalis peruviana*,* physalis-*high-protein beverage, and *Physalis-milk* beverage.

	Moisture	Carbohydrate	Protein	fats	Ash	fiber
WPC	3.58 ± 0. 34^d^	6.27 ± 0.56^d^	79.86 ± 0.14^a^	6.27 ± 0.09^a^	3.71 ± 0.02^b^	ND
*Physalis*	14.22 ± 0.88^c^	65.21 ± 1.27^a^	7.66 ± 1.34^d^	0.14 ± 0.02^d^	5.08 ± 0.94^a^	7.69 ± 1.54^a^
WHPD	70.58 ± 0. 34^a^	11.27 ± 0.56^c^	15.86 ± 0.14^c^	1.2 ± 0.09^c^	0.71 ± 0.02^d^	ND
WHPD – *Physalis*	67.07 ± 0.29^b^	13.35 ± 0.75^b^	16.17 ± 0.23^b^	1.76 ± 0.10^b^	0.91 ± 0.05^c^	0.74 ± 0.04^b^

Results expressed as means ± SEM (*n* = 3; SEM: standard error mean); ND: not detected; WPC: whey protein concentrate.

The values with a different superscript were significantly different in the same column.

WHPB: high protein beverage manufactured using the WPC.

WHPB – *Physalis*: high protein beverage manufactured using WPC and *Physalis peruviana* dried powder.

### Phytochemical properties of the prepared fermented HPD

The subsequent assessment focused on the synergistic effects of *Physalis peruviana* fortification and LAB fermentation on the phytochemical properties of the beverage. As indicated in Table 3, the total phenolic content (TPC), total flavonoid content (TFC), and antioxidant activity were significantly enhanced in the fortified and fermented HPBs relative to the non-fortified control. The HPB fortified with golden berry and fermented by *Lactobacillus plantarum* (BS-2) demonstrated the most pronounced enrichment, with the highest values for TPC (22.97 ± 0.56 mg GAE/g), TFC (97.15 ± 2.58 mg QE/g), and antioxidant activity (2.54 ± 0.31 mg TE/g). These results underscore a clear synergistic effect, where LAB fermentation likely liberates or biotransforms bound phenolic compounds from the *Physalis* matrix, thereby amplifying the antioxidant capacity. This aligns with previous findings that plant-based fortification coupled with fermentation can significantly improve the functional properties of dairy beverages^29–31,50^. The enhanced bioavailability of these bioactive compounds suggests greater potential health benefits for consumers. Golden berry supplementation significantly increased total phenolic and flavonoid contents, which enhanced antioxidant capacity as shown by ABTS and DPPH assays.

**Table 3:** Phytochemical and antioxidant activity of Raw materials, fortified, and non-fortified beverage.

	TPC (mg GAE/g DW)	TFC (mg QE/g DW)	AA as ABTS^+^ (mg TE/g DW)
WPC	0.07 ± 0.002^f^	nd	0.08 ± 0.001^g^
*Physalis*	94.27 ± 2.05^a^	254.08 ± 2.87 ^a^	4.17 ± 0.21^a^
WHPD	0.084 ± 0.009^e^	nd	0.77 ± 0.02^f^
WHPD – BS − 1	0.097 ± 0.002^e^	nd	0.92 ± 0.02^e^
WHPD – BS − 2	0.312 ± 0.027 ^d^	0.74 ± 0.05^d^	1.14 ± 0.17^d^
WHPD – BS − 3	0.264 ± 0.054 ^d^	0.39 ± 0.02^d^	1.05 ± 0.22^d^
WHPD-GB	20.28 ± 0.88^c^	86.37 ± 1.08^c^	1.84 ± 0.42^c^
WHPD-GB BS − 1	23.71 ± 0.34^b^	95.24 ± 3.14^b^	2.11 ± 0.22^b^
WHPD- GB BS − 2	22.97 ± 0.56^b^	97.15 ± 2.58^b^	2.54 ± 0.31^b^
WHPD-GB BS − 3	23.45 ± 0.77^b^	91.67 ± 3.81^b^	2.41 ± 0.27^b^

Results expressed as mean ± SEM (*n* = 3; SEM: standard error mean); ND: not detected; WPC: whey protein concentrate.

The values with a different superscript were significantly different in the same column.

TFC: total phenolic content; TFC: total flavonoid content; AA: antioxidant activity.

WHPB: high protein beverage manufactured using the WPC and plain starters strains.

WHPB-GB: high protein beverage manufactured using WPC and *Physalis peruviana* dried powder.

BS-1: *Lactobacillus pentosus* ; BS-2:*Lactobacillus plantarum* ; BS-3: *Lactobacillus paracasei*.

### Antifungal activity of the probiotic starter

**Fig. 1:**
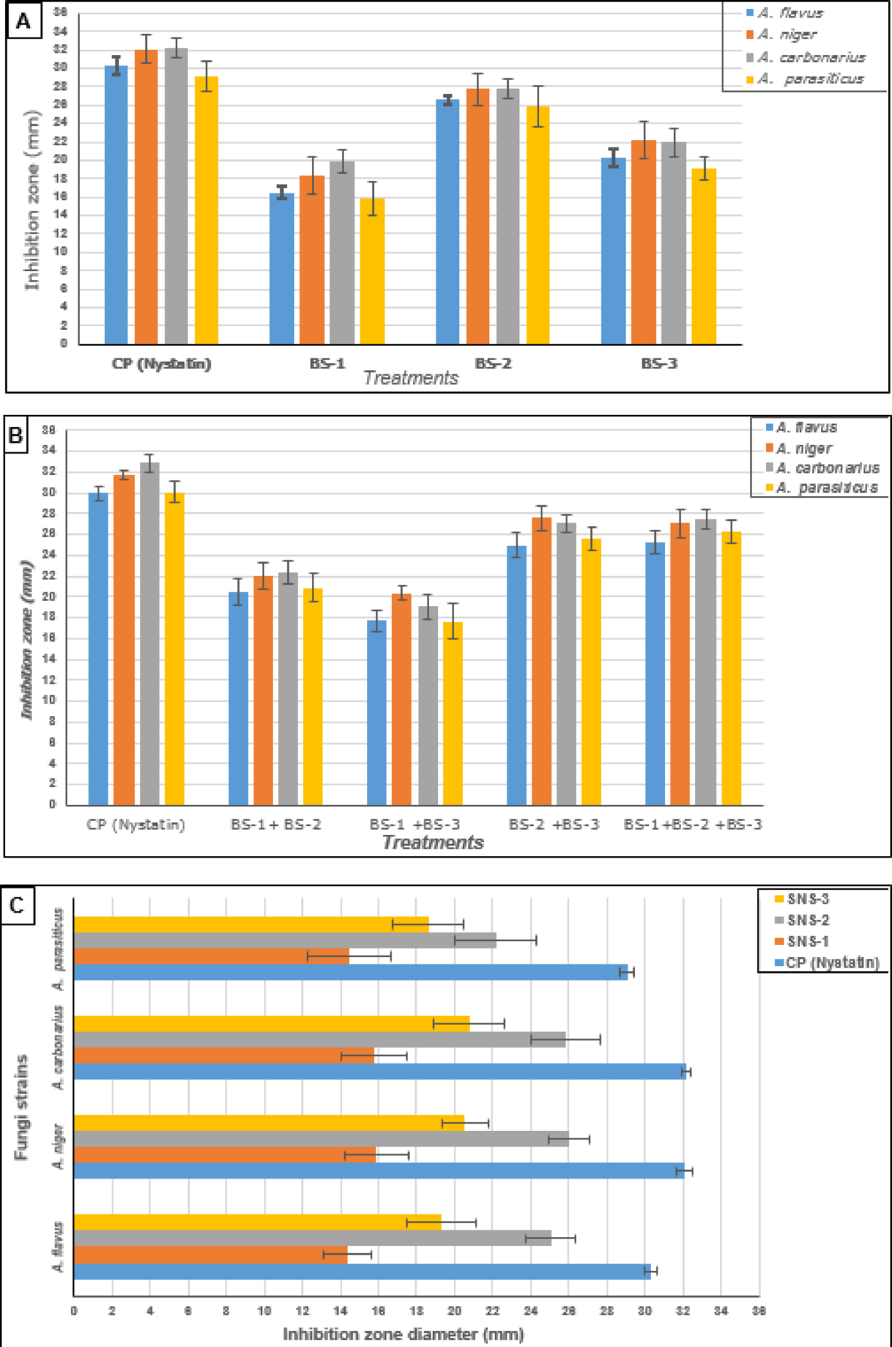
Probiotic strains’ antifungal activity (mixing or supernatant). BS-1: *Lactobacillus pentosus*; BS-2:*Lactobacillus plantarum*; BS-3: *Lactobacillus paracasei*; CP: control positive applied using nystatin antifungal drug; SNS-1: supernatant of *L. pentosus;* SNS-2: supernatant of *L. plantarum;* SNS-3: supernatant of *L. paracasei*. (**A**) antifungal activity of bacterial cells, (**B**) antifungal activity of the mix of the strain cells, (**C**) antifungal activity of probiotic strain supernatant.

A significant impact showed the antifungal activity of the new strains used for fermented HPD compared to the standard antifungal (Nystatin). The result recorded in Fig. 1 reflects the high activity of the BS–2 compared to the other strains. The individual strains’ activity against a standard antifungal compound was recorded as Nystatin > BS–2 > BS–3 > BS–1, where the last bacterial strain possesses the lowest activity (Fig. 1A). When evaluating the mixture of bacterial strains, the best results were obtained for the mixture (BS–2 + BS–3) in terms of antifungal activity, similar effects were observed for the mixture of three strains (BS-1 + BS-2 + BS-3) (Fig. 1B). The mixture strains (BS–1 + BS–3) recorded the lowest antifungal effect against the four applied fungal strains. It was noticed in the two experimental trials that *A. parasiticus* possessed a less sensitive fungal response to the bacterial strain application (Fig. 1A and B). The antifungal activity of bacterial supernatants was evaluated against the four mycotoxigenic strains. A high inhibition effect was recorded for the SB–2 bacterial strain supernatant (Fig. 1C). The lowest inhibition was recorded for the collected supernatant from the SB–1 bacterial strain growth.

The antifungal effect of probiotic strains was reported for several bacterial strains^32^. These strains include *Lactobacillus spp.*, *Bifidobacterium spp*., and *Streptococcus spp*., where the inhibition ratio reached 51% of total fungal growth. Other probiotic strains of *L. rhmanosus* and *L. paracasei* were also reported to possess significant antifungal activity^33^. The previous studies pointed out the impact of secondary metabolites as antifungal agents in the growth of fungal media^28^. The growth of probiotic bacterial strains may produce several antifungals in media, such as organic acids and bacitracin^21,22^. The impact of these metabolites against toxigenic fungal strains was recorded in several investigations^21,34^. The antifungal activity of bacterial strains against aflatoxigenic fungal strains was shown by a highly significant impact to apply in food or feed production^35^. Bacterial strains of probiotics were also reported to reduce mycotoxin availability^36^. The effect of probiotic strains of bacteria may be extended to affect the hazard in the gastrointestinal tract of the consumers^37^.

The high antifungal activity of *L. plantarum* (BS-2) is likely due to its well-documented capacity to produce potent antifungal metabolites, such as specific bacteriocin^22,34^ and organic acids with efficiency against toxigenic fungal strains^11,38^. In contrast, *L. pentosus* (BS-1) is known to produce extracellular bio-emulsifiers or polysaccharides, particularly in media enriched with cellulose^39^. The fruit powder used in our study may not have provided optimal substrates for polysaccharide production by BS-1, potentially explaining its lower antifungal activity compared to BS-2.

### Fungal growth inhibition

The inhibition effect of bacterial supernatant was recorded, as shown in Table 4. The documented impact against the aflatoxin-producing strain of *A. parasiticus* was significant in the present experiment. The inhibition ratio (%) was recorded as 53.80 ± 0.41, 44.18 ± 0.27, and 30.65 ± 0.73 for the supernatant collected from BS-1, BS-2, and BS-3 strains. On the other hand, aflatoxin reduction was also examined in the liquid media growth. A valuable reduction was shown regarding the secreted four aflatoxin types (AFB_1_, AFB_2_, AFG_1_, and AFG_2_). Compared to the control, the BS-2 supernatant applied in the growth media can reduce aflatoxins to the lowest secretion level among the several applied supernatants (Table 4**)**, where Aflatoxin B_2_ was recorded as not detected by BS-2 supernatant application. The reduction of these compounds is recorded as the lowest value for applying BS-1 supernatant in the *A. parasiticus* strain^28^. The inhibition of the growth of toxigenic fungal strains was reported earlier by Shehata et al. (2019 ). The results showed high inhibition of the *L. plantarum* strain, with reduced aflatoxin production^22^. Similarly, the evaluation of bacterial supernatant against mycotoxigenic fungal strains was reported by Mogahed Fahim et al. (2021). The tested bacterial strains *Lactobacillus plantarum* RM1 and *Lactobacillus paracasei* KC39 inhibited the growth of the *Aspergillus* fungus, and their supernatants reduced aflatoxin production. The reduction of aflatoxins was also recorded for the metabolite toxin of aflatoxin M_1_.

**Table 4:** Bacterial extract effect on *A. parasiticus* producing strain for growth rate and aflatoxins production.

	Mycelial weight (g)	Inhibition ratio (%)	AFB_1_ (ng/mL)	AFB_2_ (ng/mL)	AFG_1_ (ng/mL)	AFG_2_ (ng/mL)
Control	5.97 ± 0.24^a^	–	191.74 ± 3.51^a^	158.66 ± 2.81^a^	189.37 ± 4.08^a^	147.29 ± 2.69^a^
BS-1	3.97 ± 0.37^b^	30.65 ± 0.73^c^	107.66 ± 4.84^b^	113.66 ± 3.84 ^b^	101.54 ± 4.28 ^b^	102.51 ± 4.16 ^b^
BS-2	2.65 ± 0.22^d^	53.80 ± 0.41^a^	27.59 ± 1.56^c^	nd	21.18 ± 1.97 ^d^	12.64 ± 3.47 ^cd^
BS-3	3.11 ± 0.14^c^	44.18 ± 0.27^b^	74.87 ± 3.67 ^c^	93.16 ± 3.89 ^c^	79.66 ± 5.02 ^c^	69.47 ± 3.67 ^c^

Results expressed as means ± SEM (*n* = 3; SEM: standard error mean); ND: not detected; WPC: whey protein concentrate.

The values with a different superscript were significantly different in the same column.

BS-1: *Lactobacillus pentosus* ; BS-2:*Lactobacillus plantarum ;* BS-3: *Lactobacillus paracasei.*

AFB_1_: aflatoxin B_1_; AFB_2_: aflatoxin B_2_; AFG_1_: aflatoxin G_1_; and AFG_2_: aflatoxin G_2_.

### Determination of viscosity changes

Beyond phytochemical enhancement, it was evaluated the impact of bacterial strains and Physalis fortification on beverage viscosity—a critical parameter for consumer acceptability, product stability, and shelf-life^4,40,41^.

**Fig. 2:**
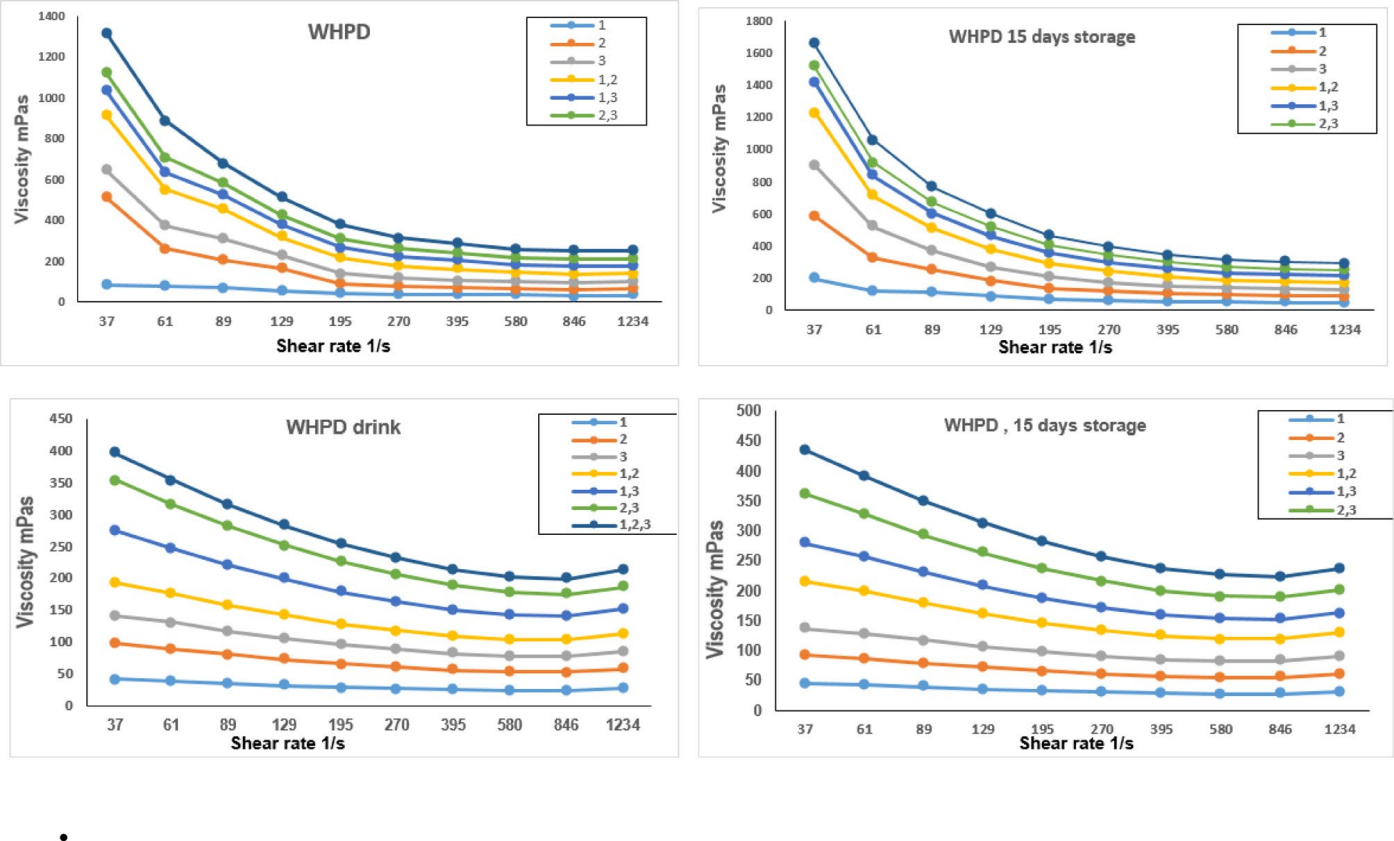
Changes in beverage rheology characteristics manufactured using three bacterial strains (individuals or mixes) with or without golden berry powder insertion. HPB: high protein beverage without fortification; FHPB: high protein beverage fortified with *physalis powder*.

Figure 2 illustrates that the fortification and fermentation of *Physalis* with LAB strains, both individually and in combinations, resulted in a significant increase in the viscosity of the HPBs. Beverages inoculated with the three-strain mix exhibited the highest apparent viscosity, indicating improved protein-polysaccharide interactions and structural stability. The results illustrated significant differences in the viscosity between beverages. It was observed that the bacterial strains had a significant effect on the viscosity of the beverages. The highest viscosity of the beverage was observed when using three strains of the tested bacteria. The results are in line with the studies conducted by^42 and^^43^, which indicated that the addition of plant-derived polysaccharides and bacterial metabolites during fermentation can alter the rheological properties of dairy beverages, potentially enhancing mouthfeel and product stability. Moreover, the shelf life of the high-protein beverage could be linked to its viscosity values. Due to the contamination occurring in dairy beverages, the harmful microorganisms secrete enzymes that are thick^43^.

This result also mentioned the viscosity and shear stress rheological properties for all analyzed samples subjected to the plain beverage and the introduced bacterial strains, regardless of the presence of GB. The rheological properties (viscosity and shear stress) of all beverages, with or without GB, were influenced by the bacterial strains used (Fig. 2). The addition of GB powder itself induced significant rheological changes, altering the shear rate and stress profiles. Furthermore, fermentation with the novel bacterial strains, particularly the three-strain mix, caused substantial changes, likely due to protein-polysaccharide interactions and metabolic alterations affecting protein denaturation, as indicated by the pronounced changes in flow curves. The GB powder contains bioactive components, such as polysaccharides, which can play a health-benefit role in vivo against food hazards^44^. The HPD with the GB powder demonstrated higher viscosity compared to the standard HPD while exhibiting a consistent trend for the inoculation strains employed. The results indicate higher concentrations of solids, proteins, and fats in Physalis peruviana powder.

Similarly, bioactive saccharides of the GB are a suitable substrate for the bacterial starter to increase the acidity and change the protein rheology. Again, the author evaluated these strains previously, and they could produce polysaccharides in their metabolism^16^. This point may explain the changes in the plain’s product rheology and for the GB beverage.

### Sensory evaluation of the HPD

**Fig. 3:**
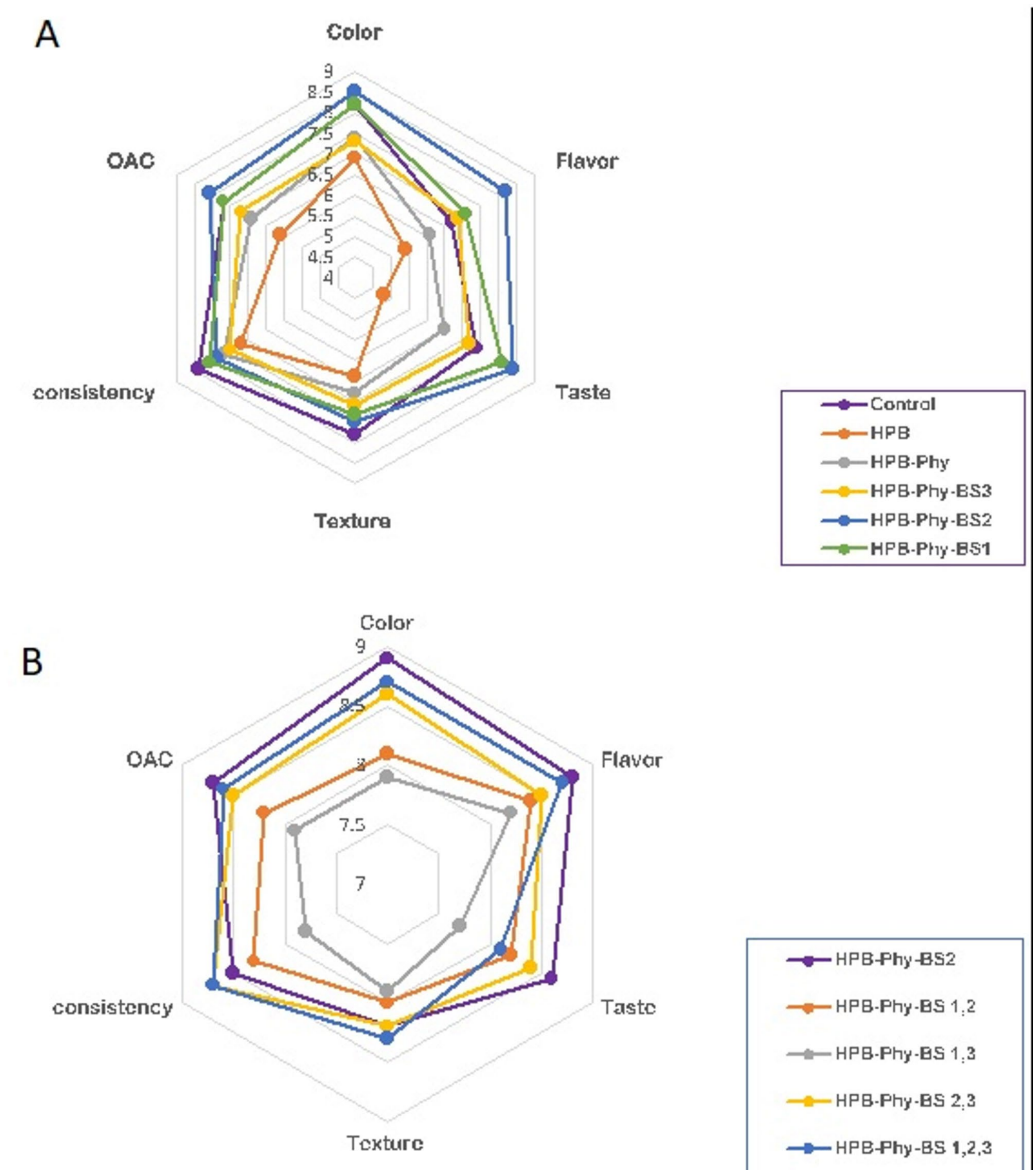
Sensory evaluation of the fermented high protein beverage (HPB) manufactured use of individual or mixed strains. Changes in acceptability of fortified beverages with *Physalis* prepared using individual starter strains (**A**) and those prepared using mixed starter strains of bacteria. BS1: *Lactobacillus pentosus*; BS2: *Lactobacillus plantarum*; BS3: *Lactobacillus paracasei*. Control: HPB with traditional starter; HPB-Phy: high protein beverage with phyallis; HPB-Phy-BS1: high protein beverage with phyallis and BS1 strain; HPB-Phy-BS2: high protein beverage with phyallis and BS2 strain; HPB-Phy-BS3: high protein beverage with phyallis and BS3 strain.

The sensory evaluation was conducted according to institutional ethical guidelines. All panelists provided written informed consent prior to participation. The sensory protocol was reviewed and approved by the Institutional Research Ethics Committee of the National Research Centre (Approval given NRC-SENS/2023/01). To ensure the consumer acceptability of the fortified HPBs, a sensory evaluation was performed. Sensory evaluation of the HPD (with or without *Physalis)* was determined, and the results were recorded in Fig. 3. Firstly, *Physalis*-HDP, manufactured using an individual strain of applied bacterial starters, was compared to the control (commercial fermented milk). The Physalis-fortified HPB fermented with *L. plantarum* (BS-2) received the highest overall acceptability scores compared to other formulations (Fig. 3A). Compared to the HPD performed by a bacterial mix, *Physalis-*BS 2 is still the more acceptable product preferred by the panelists (Fig. 3B). The positive sensory perception of the BS-2-fermented beverage underscores the dual benefit of improved nutritional and safety attributes alongside enhanced organoleptic properties. These results are consistent with previous findings by Nam^45^ or Cho et al.^46^, highlighting the important role of specific probiotic strains in modulating flavor and texture profiles in functional beverages.

### Extension of the HPD shelf life using the bacterial strains

**Fig. 4:**
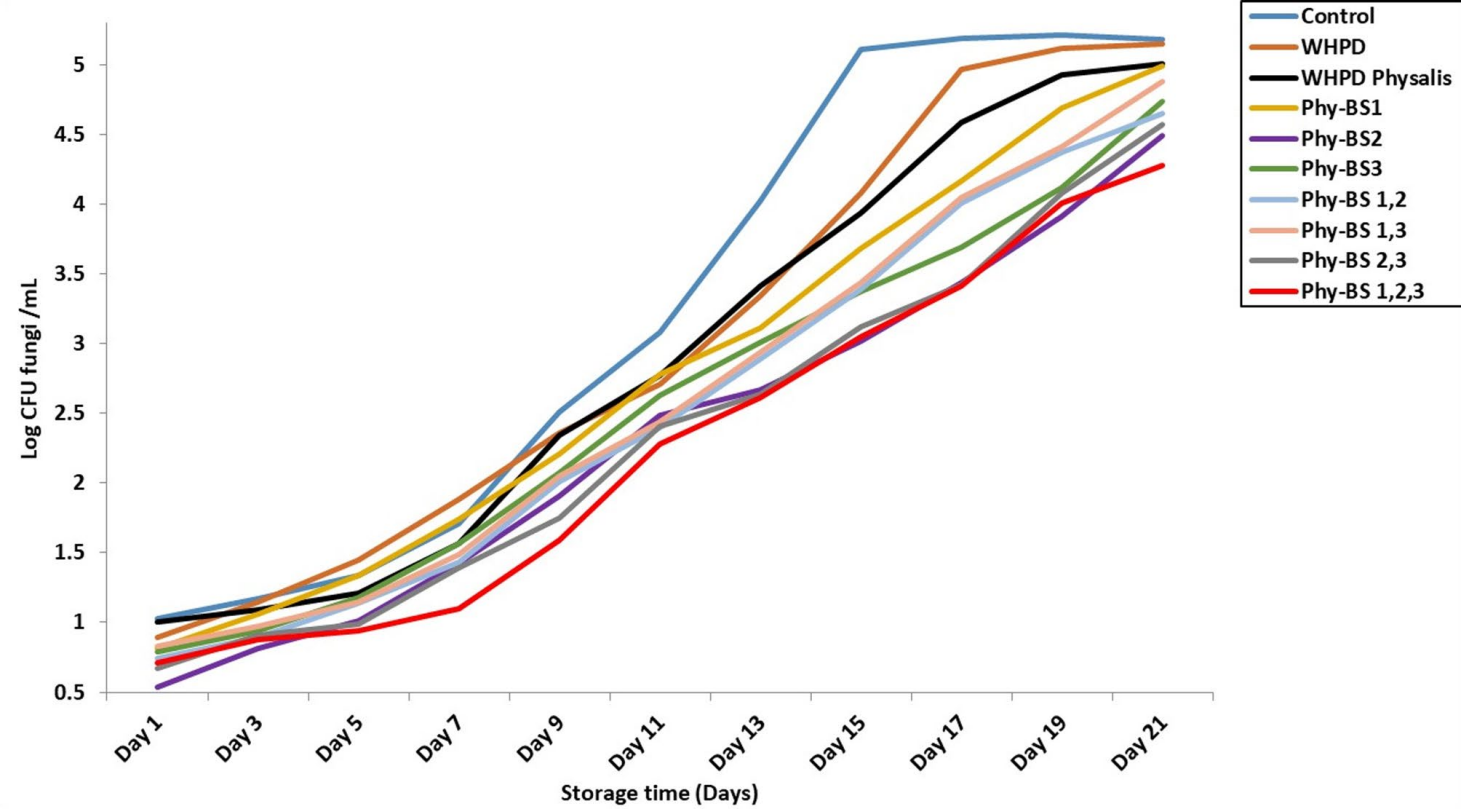
Shelf life evaluation and storage extension for the high protein beverage (HPG) manufactured using individual and mixed starters of bacterial strains. HPG: high protein beverage without fortification; HPB-Phy: high protein beverage fortified with *Physalis* (golden berry). BS1: *Lactobacillus pentosus*; BS2: *Lactobacillus plantarum*; BS3: *Lactobacillus paracasei*. Control: HPB with traditional starter; HPB-Phy: high protein beverage with phyallis; HPB-Phy-BS1: high protein beverage with phyallis and BS1 strain; HPB-Phy-BS2: high protein beverage with phyallis and BS2 strain; HPB-Phy-BS3: high protein beverage with phyallis and BS3 strain.

To assess market potential, it was evaluated the ability of the bacterial strains to extend shelf-life under simulated fungal contamination. As shown in Fig. 4, beverages fermented with the three-strain mix or with BS-2 alone maintained the lowest fungal counts (CFU/mL) throughout the 21-day storage at 4 °C. The HDP produced with the bacterial mix showed the greatest resistance to fungal growth, followed closely by the beverage fermented solely with *L. plantarum* (BS-2). The smaller increase in log CFU/mL values in these treatments demonstrates the efficacy of the bacterial strains in enhancing product shelf-life. The significant variation in final log CFU/mL values between treatments highlights the differences in the shelf-life extending capability of the various bacterial strains. The decrease in fungal growth is due to the synergistic actions of LAB and Physalis bioactives, together with the generation of bioactive peptides and organic acids that possess antifungal and anti-aflatoxigenic properties. The bacterial efficiency to resist the microbial changes in the product may be explained by the bacterial metabolites excreted in their media^47^. Dairy fermentation results from the activity of the proteolytic LAB or their proteolytic hydrolysis enzymes, which is the step for active peptide production^48^. The bioactive peptides produced by the LAB strains were recorded as having antifungal activities^49^. LAB strains produce organic acids in media that reduce the contamination chance^50^. These acids play an antifungal functionality, also an anti-aflatoxigenic activity^31^. These forms of LAB activities can explain the extension recorded in the product shelf life and the decrease in the log CFU infection for the inoculated fungi.

The high antifungal activity observed for the BS-2 strain (*Lactobacillus plantarum*) may be attributed to its genus-specific capacity to produce various antifungal metabolites, such as organic acids, cyclic peptides, and bacteriocins^8,51^. Previous studies have shown that *L. plantarum* strains are capable of producing organic acids such as lactic, acetic, and phenyllactic, as well as antifungal cyclic peptides, which collectively contribute to their broad-spectrum antifungal activity^52,53^. These metabolites inhibit fungal growth via mechanisms including disruption of cell membranes, reduction of pH, and chelation of essential minerals necessary for fungal metabolism. Guimarães et al. (2018) demonstrated that lactic acid and phenyllactic acid produced by L. plantarum inhibit the growth of *Aspergillus* and *Penicillium* species^53^. Our future part of the study will concentrate on characterizing the specific antifungal metabolites generated by BS-2 within the beverage matrix and examining their interactions with mycotoxigenic fungi for the in-vivo model.

## Conclusion

This research introduces a new approach to improving the functional and microbiological quality of high-protein dairy beverages by co-applying *Physalis peruviana* powder and specific probiotic LAB strains. The fortified and fermented formulations demonstrated enhanced phytochemical content, antioxidant capacity, and antifungal activity relative to non-fortified controls. *Lactobacillus plantarum* (BS-2) was identified as the most effective strain, exhibiting significant inhibition of *Aspergillus* sp., growth and aflatoxin biosynthesis, particularly achieving complete suppression of AFB2. Fortified formulations exhibited improved rheological properties, with increased viscosity associated with greater product stability and sensory acceptance. LAB act as both bio-preservatives and nutritional enhancers; their capacity to reduce fungal growth and extend shelf life supports their industrial use in clean-label, health-oriented beverages. The observed decrease in mycotoxins, along with positive organoleptic characteristics, indicates a feasible substitute for chemical preservatives in dairy products. Future research should concentrate on the molecular characterization of antifungal metabolites generated by LAB in the presence of *Physalis* compounds, along with the validation of these effects in in vivo gastrointestinal models and pilot-scale production environments. While the results under laboratory conditions are highly promising, further extensive durability and stability tests under industrial storage conditions, along with pilot-scale production trials and In-vivo studies, are necessary to fully validate this approach as an effective alternative to chemical preservatives in commercial applications. This study promotes the idea of multifunctional fermented beverages, highlighting their relevance to food safety, nutrition, and consumer health.

## Supplementary Information

Below is the link to the electronic supplementary material.

Supplementary Material 1

## Data Availability

The datasets used and/or analyzed during the current study available from the corresponding author on reasonable request.
