# Supplementary material for: Synergistic antifungal and anti-aflatoxigenic effects of lactic acid bacteria and golden berry in a functional high-protein beverage
Source: Sci Rep. 2025 Dec 8;15:43378. doi: 10.1038/s41598-025-24160-y (PMC12689706; doi:10.1038/s41598-025-24160-y)
Supplement: Supplementary file 1 — Supplementary Material 1 [file 41598_2025_24160_MOESM1_ESM.pdf]

Supplementary materials

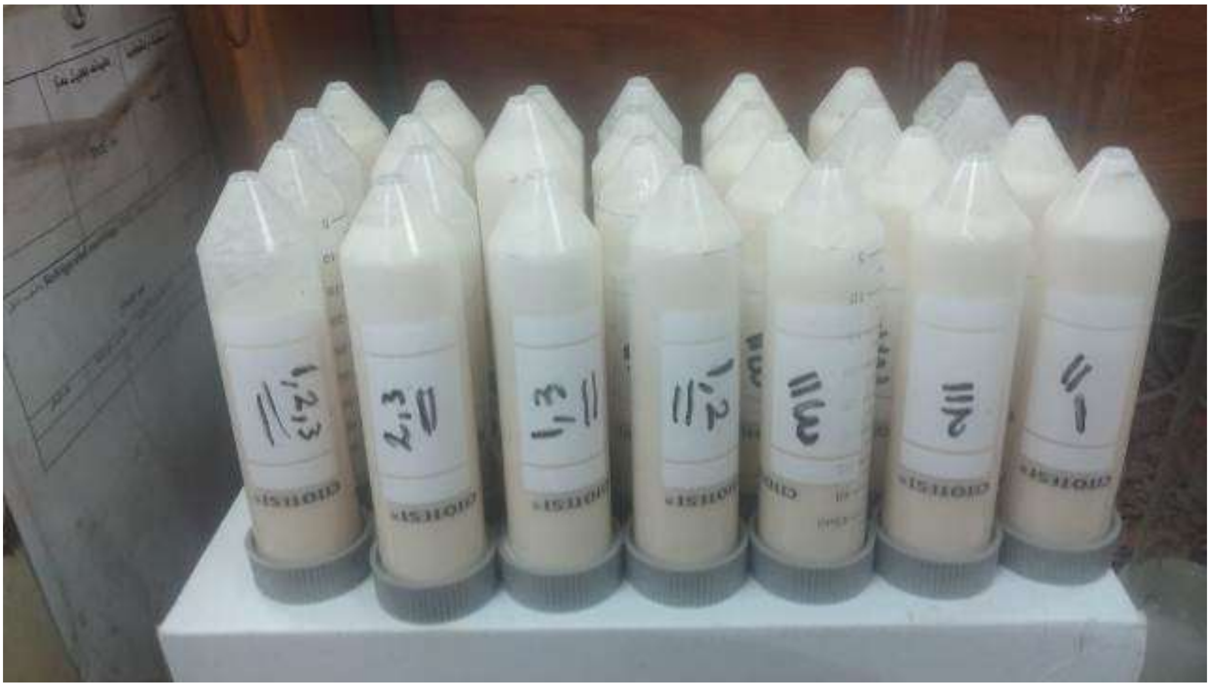

Figure S1: Milk beverage fortified with golden berry powder and fermented using 3 strains of probiotic starters (individual or integrated)

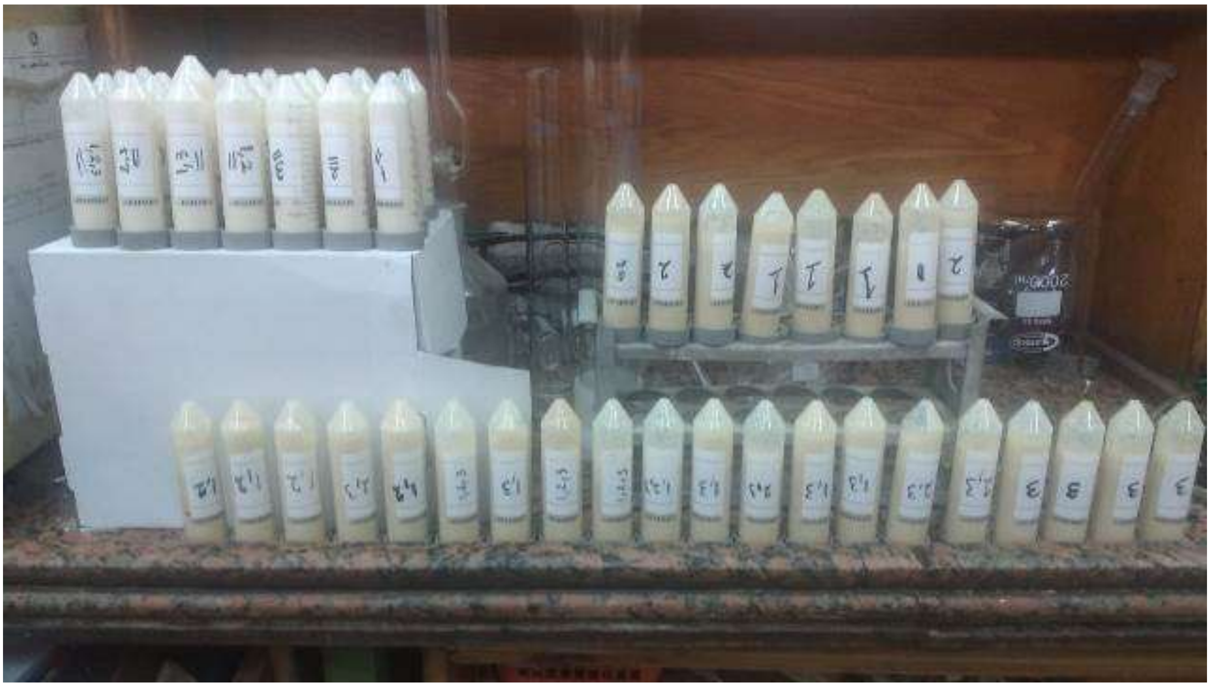

Figure S2: Milk beverage (planed or fortified with golden berry powder) that fermented using 3 strains of probiotic starters (individual or integrated)

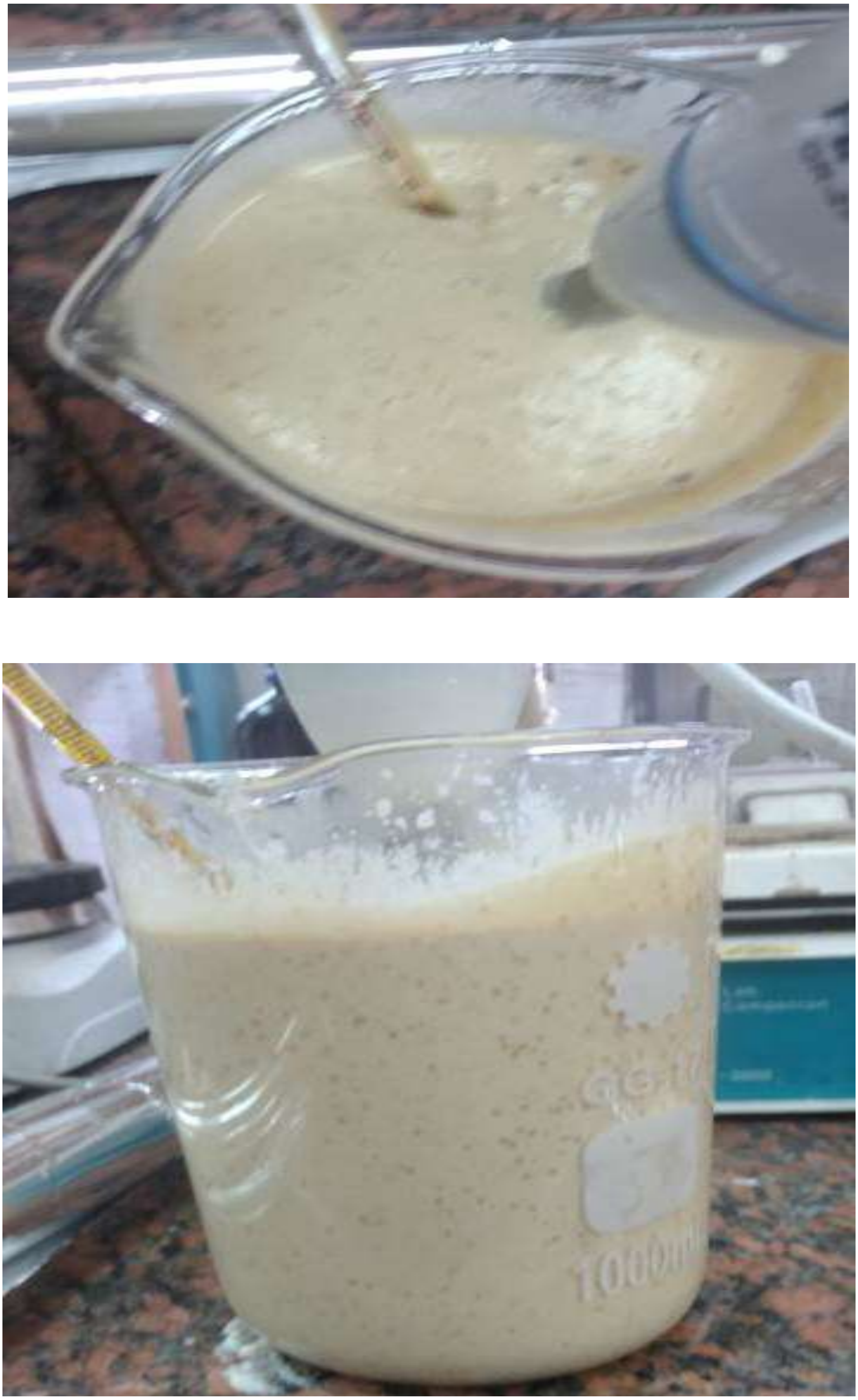

Figure S3: Prepared samples of the fermented milk beverage fortified with golden berry powder
